# The Association Between Vaginal Microbiota Dysbiosis, Bacterial Vaginosis, and Aerobic Vaginitis, and Adverse Pregnancy Outcomes of Women Living in Sub-Saharan Africa: A Systematic Review

**DOI:** 10.3389/fpubh.2020.567885

**Published:** 2020-12-10

**Authors:** Naomi C. A. Juliana, Meghan J. M. Suiters, Salwan Al-Nasiry, Servaas A. Morré, Remco P. H. Peters, Elena Ambrosino

**Affiliations:** ^1^Department of Genetics and Cell Biology, Faculty of Health, Medicine and Life Sciences, Research School GROW (School for Oncology & Developmental Biology), Institute for Public Health Genomics, Maastricht University, Maastricht, Netherlands; ^2^Department of Obstetrics and Gynecology, GROW School of Oncology and Developmental Biology, Maastricht University Medical Center (MUMC), Maastricht, Netherlands; ^3^Laboratory of Immunogenetics, Department of Medical Microbiology and Infection Control, Amsterdam UMC, Amsterdam, Netherlands; ^4^Department of Medical Microbiology, University of Pretoria, Pretoria, South Africa; ^5^Department of Medical Microbiology, School for Public Health and Primary Care (CAPRHI), Maastricht University, Maastricht, Netherlands; ^6^Research Unit, Foundation for Professional Development, East London, South Africa

**Keywords:** vaginal microbiota (VMB), bacterial vaginosis (BV), anaerobic vaginitis (AV), vaginal dysbiosis, pregnancy outcomes, sub-saharan africa, microbiome, systematic review

## Abstract

**Background:** Previous studies have described the association between dysbiosis of the vaginal microbiota (VMB) and related dysbiotic conditions, such as bacterial vaginosis (BV) and aerobic vaginitis (AV), and various adverse pregnancy outcomes. There is limited overview of this association from countries in sub-Saharan Africa (SSA), which bear a disproportionally high burden of both vaginal dysbiotic conditions and adverse pregnancy outcomes. This systematic review assesses the evidence on the association between VMB dysbiosis, BV, and AV, and late adverse pregnancy outcomes in women living in SSA.

**Methods:** The Preferred Reporting Items for Systematic Review and Meta-Analysis Statement (PRISMA) guidelines were followed. Three databases [PubMed, Embase (Ovid), and Cochrane] were used to retrieve observational and intervention studies conducted in SSA that associated VMB dysbiosis, BV, or AV and preterm birth/labor/delivery, preterm rupture of membranes (PROM), low birthweight, small for gestational age, intrauterine growth restriction, intrauterine infection, intrauterine (fetal) death, stillbirth, perinatal death, or perinatal mortality.

**Results:** Twelve studies out of 693 search records from five SSA countries were included. One study identified a positive association between VMB dysbiosis and low birthweight. Despite considerable differences in study design and outcome reporting, studies reported an association between BV and preterm birth (7/9), low birthweight (2/6), PROM (2/4), intrauterine infections (1/1), and small for gestational age (1/1). None of the retrieved studies found an association between BV and pregnancy loss (5/5) or intrauterine growth retardation (1/1). At least two studies support the association between BV and PROM, low birthweight, and preterm birth in Nigerian pregnant women. No reports were identified investigating the association between AV and late adverse pregnancy outcomes in SSA.

**Conclusion:** Two of the included studies from SSA support the association between BV and PROM. The remaining studies show discrepancies in supporting an association between BV and preterm birth or low birthweight. None of the studies found an association between BV and pregnancy loss. As for the role of VMB dysbiosis, BV, and AV during pregnancy among SSA women, additional research is needed. These results provide useful evidence for prevention efforts to decrease vaginal dysbiosis and its contribution to adverse pregnancy outcomes in SSA.

## Introduction

Improving maternal and perinatal health is one of the biggest medical and public health challenges in sub-Saharan Africa (1–3). Infectious diseases are one of the multiple causes of adverse pregnancy outcomes (1, 4, 5). To improve maternal health and reduce adverse pregnancy outcomes in Africa, various prevention measurements to lower malaria transmission, mother-to-child human immunodeficiency virus (HIV) transmission, and improve symptomatic sexually transmitted infections screening have been implemented during antenatal and perinatal consultations (6, 7).

Over the past decades, there have been increasing discussions on whether vaginal dysbiosis (defined here as not dominated by lactobacilli), the imbalance of the vaginal commensal bacterial communities (microbiota), influences pregnancy outcomes and should be monitored antenatally (8, 9). In sub-Saharan Africa, there is a particularly high burden of vaginal-dysbiosis-related conditions (10).

Generally, the vaginal microbiota (VMB) consists of commensal microorganisms that exist in a dynamic, complex, and mutually beneficial relationship with the host (11). Healthy VMB are crucial to the lower female reproductive tract. VMB contains a predominance of hydrogen-peroxide-producing *Lactobacillus* species that contribute to an immunological balance and therefore support a healthy reproductive tract (12–14). However, many factors can influence the VMB composition, such as nutrition, sexual and hygienic practices, ethnicity, and hormonal fluctuations during menstruation or pregnancy (15). Bacterial vaginosis (BV) is one of the most common vaginal dysbiotic conditions worldwide. It affects 10–30% of women at any given time, with a higher prevalence in sub-Saharan African women compared to other world regions (16–18). However, some populations in parts of Africa have low BV prevalence (6–8%), as for instance reported in Burkina Faso, while in South Africa, studies have reported high BV prevalence (34–58%) (18). This vaginal condition occurs when there is a shift from a *Lactobacillus*-dominant VMB to a more diverse VMB, rich in anaerobic bacteria with, for instance, species from *Gardnerella, Prevotella*, and *Atopobium* genera (19). BV prevalence varies with ethnicity, socioeconomic conditions, and gestational age. BV is also common during pregnancy and multiple independent studies have observed an association between BV and preterm birth (PTB), as well as miscarriage, maternal infection, and low birth weight (LBW) (17, 19, 20). Two decades ago, two meta-analyses confirmed that BV during pregnancy increased PTB risk by >2-fold (21, 22). However, compared to PTB, less research had been conducted for other obstetric outcomes (23). Aerobic vaginitis (AV) is another vaginal dysbiotic condition characterized by an abnormal VMB composed mostly of commensal aerobic microorganisms of intestinal origin, typically *Escherichia coli, Staphylococcus aureus*, coagulase-negative staphylococci such as *Staphylococcus epidermidis*, group B *Streptococcus* (*Streptococcus agalactiae*), and *Enterococcus faecalis* (24–29). The prevalence of AV is between 5 and 10.5% among symptomatic non-pregnant women and between 4 and 8% among pregnant women (26, 29). AV has also been associated with a higher risk of spontaneous miscarriage, preterm prelabor rupture of membranes, chorioamnionitis, and preterm delivery (PTD) (30–32). Increasing evidence is suggesting that VMB dysbiosis is associated with various adverse pregnancy outcomes, such as an increased risk of postabortion infection, early and late miscarriage, histological chorioamnionitis, postpartum endometritis, premature rupture of membranes (PROM), and preterm birth (PTB) (20, 33–42). A significant decrease in VMB richness (number of species), evenness (relative abundance of each species), and diversity (the overall richness and evenness of species) during pregnancy is associated with PTB (43). Furthermore, individual vaginal colonization with bacterial pathobionts (symbionts with pathogenic potential, like streptococci, staphylococci, or *Enterobacteriaceae* species) during pregnancy can associate with adverse pregnancy outcomes (44, 45). Son et al. (46) reported that a *Klebsiella pneumoniae*-dominant VMB might be associated with PTB before 28 weeks of gestational age (GA), *Lactobacillus iners* with PTB before 34 weeks of GA, and *S. agalactiae* with late miscarriage (46). In contrast, VMB dominated by *L. crispatus* have not been associated with PTB (46–48).

It has been proposed that the increased risk of adverse reproductive and pregnancy outcomes might be attributable to BV-related bacterial species rather than BV itself. In the context of *in vitro* fertilization (IVF) studies, patients with high incidence of *Gardnerella, Atopobium*, and *Prevotella* failed to become pregnant after embryo transfer or experienced miscarriage (49, 50). Indeed, in early pregnancy, the reduction in lactobacilli and the overgrowth of *Gardnerella, Atopobium*, and *Prevotella* genera disrupt the microbial homeostasis, hindering embryo implantation (49). Other studies also observed that anaerobe bacteria influence the late stages of pregnancy. For instance, DiGiulio et al. reported a strong association between PTB and the presence of *Gardnerella* and *Ureaplasma*, both BV-related bacteria (51). Interestingly, these last findings were not supported by a larger study conducted by Romero et al. (52). The small sample sizes and different study populations may explain disparities between the two studies. The former study included mostly Caucasian women (60%), whereas the latter were mostly African American women (86%) (51, 52). The inherent differences in the VMB between women of different ethnic backgrounds have been first reported in the ground-breaking comparative study conducted by Ravel et al. (53). The study observed that healthy asymptomatic women of African ancestry living in North America had a more diverse and less *Lactobacillus*-rich VMB than women from other ethnic backgrounds living in the same environment (53). Increasing evidence on the topic suggests that non *Lactobacillus*-dominant VMB might not correspond to clinically relevant dysbiosis in asymptomatic healthy non-Caucasian women (54).

Nonetheless, women of African ancestry living in the United States of America, have a higher probability of PTB attributable to BV and vaginal inflammation, compared to Caucasian or Hispanic women living in the same area (55, 56).

To date, most VMB profiling studies, especially those in pregnancy, have been conducted among Caucasian in Europe or North America. There is increasing research interest in understanding how VMB composition and related conditions might contribute to, and offer diagnostic potential for, adverse pregnancy outcomes. There is a knowledge gap on VMB diversity or vaginal conditions and associated pregnancy outcomes in settings with high rates of pregnancy complications and with populations of non-Caucasian ancestries, such as sub-Saharan Africa.

This study aimed to systematically review evidence on the association between VMB dysbiosis, BV, and AV, and late adverse pregnancy outcomes in women living in sub-Saharan Africa. The results provide information for the improvement of risk assessment and the effectiveness of clinical practices during pregnancy in sub-Saharan Africa.

## Methods

This review was conducted in line with the Preferred Reporting Items for Systematic Review and Meta-Analysis Statement (PRISMA) guidelines. Supplementary Table 1 contains the corresponding completed PRISMA Checklist form (57). Articles were retrieved from PubMed/Medline, Embase (Ovid), and the Cochrane Databases.

### Eligibility Criteria

All original studies that analyzed the association between VMB dysbiosis, BV, and AV, and late adverse pregnancy outcomes in sub-Saharan African countries were eligible for inclusion. Studies were only included if, at the time of sampling, the participants were pregnant. Studies that diagnosed BV via Gram staining or Amsel clinical criteria were included (58–60). For AV, studies that diagnosed AV via wet mounts of fresh vaginal discharge, as Donders et al. reported, or followed clinical features and wet smear microscopy diagnosis, as Tempera et al. proposed, were included (24, 61). Non-*Lactobacillus-*dominant VMB were considered indicative of VMB dysbiosis in this review. Studies were not selected based on participants' characteristics. All observational and intervention study designs were eligible for inclusion, except case reports, case series, surveys, and other (systematic) reviews. Studies were excluded if they were not performed on human subjects, did not occur among women from the sub-Saharan African region, or if they observed pregnancy outcomes that occurred solely before 20 weeks of GA. No language or date restriction was used.

### Type of Outcome Measures

Various late adverse pregnancy outcomes in relation with BV, AV, or VMB dysbiosis in pregnant women from sub-Saharan Africa were selected. Studies that observed pregnancy outcomes that occurred at 20 weeks of GA, or after, were classified as late pregnancy outcomes. These outcomes were PTB, preterm labor (PTL), PTD, PROM, LBW, late miscarriage, small for gestational age (SGA), intrauterine growth restriction (IUGR), intrauterine infection, including chorioamnionitis, intrauterine death (IUD), intrauterine fetal death (IUFD), perinatal death (PND), stillbirth, and perinatal mortality. The outcomes had to be from the current pregnancy.

### Literature Search

PubMed, Embase (Ovid), and Cochrane database searches were independently conducted up to May 7th, 2020. Medical subject heading (MeSH) and Embase subject heading (Emtree) terms, free text terms, and the combination of keywords used for these searches can be found in Supplementary Table 2. The corresponding keywords were “vaginal microbiome,” “vaginal microbiota,” “vaginal dysbiosis,” “bacterial vaginosis,” “aerobic vaginitis,” “abnormal vaginal flora,” “Africa,” “sub-Saharan Africa,” “pregnant women,” “pregnancy,” and “pregnancy outcome.” In order to ensure that no studies were missed, additional searches were performed, in which the keywords “Africa” or “sub-Saharan Africa” were changed into the name of each sub-Saharan African country, based on the World Bank Organization database (62).

### Selection of Studies

Titles and abstracts were assessed independently by two reviewers (NJ and MS). Thereafter, selected articles were fully read to determine if they met the inclusion criteria. If there were any uncertainties to include an article, a third reviewer (EA or RP) was consulted to achieve consensus. Conference abstracts and the bibliographies of articles, also if excluded but relevant, were examined to retrieve further potential articles containing unique data.

### Data Extraction

For evidence on the contribution of BV, AV, or VMB diversity to pregnancy outcomes, the full set of related results from each article was extracted. The odds ratio (OR) and 95% confidence intervals (CI) were extracted separately from each study. Additionally, the OR, 95 CI%, or *P*-values were calculated from the reported data if they were not mentioned in the original article (63–65). Findings of studies where no ORs were mentioned or could not have been calculated were also reported.

### Risk of Bias in Individual Studies

To assess the methodological quality of the different individual studies, the Joanna Briggs Institute (JBI) Critical Appraisal Tool was used (66). Different JBI checklists for specific study designs were used to determine their potential risk of bias (Supplementary Table 3). Questions that were answered with “yes” were assigned 1 point; “unclear or only partly discussed,” 0.5 points; and “no,” 0 points. The risk of bias of individual studies was determined as “low risk of bias” if the study scored at least 70%, “moderate risk of bias” if the study score was between 50 and 69%, and “high risk of bias” if the study scored 49% or less (66–68).

## Results

### Study Selection

**Figure 1:**
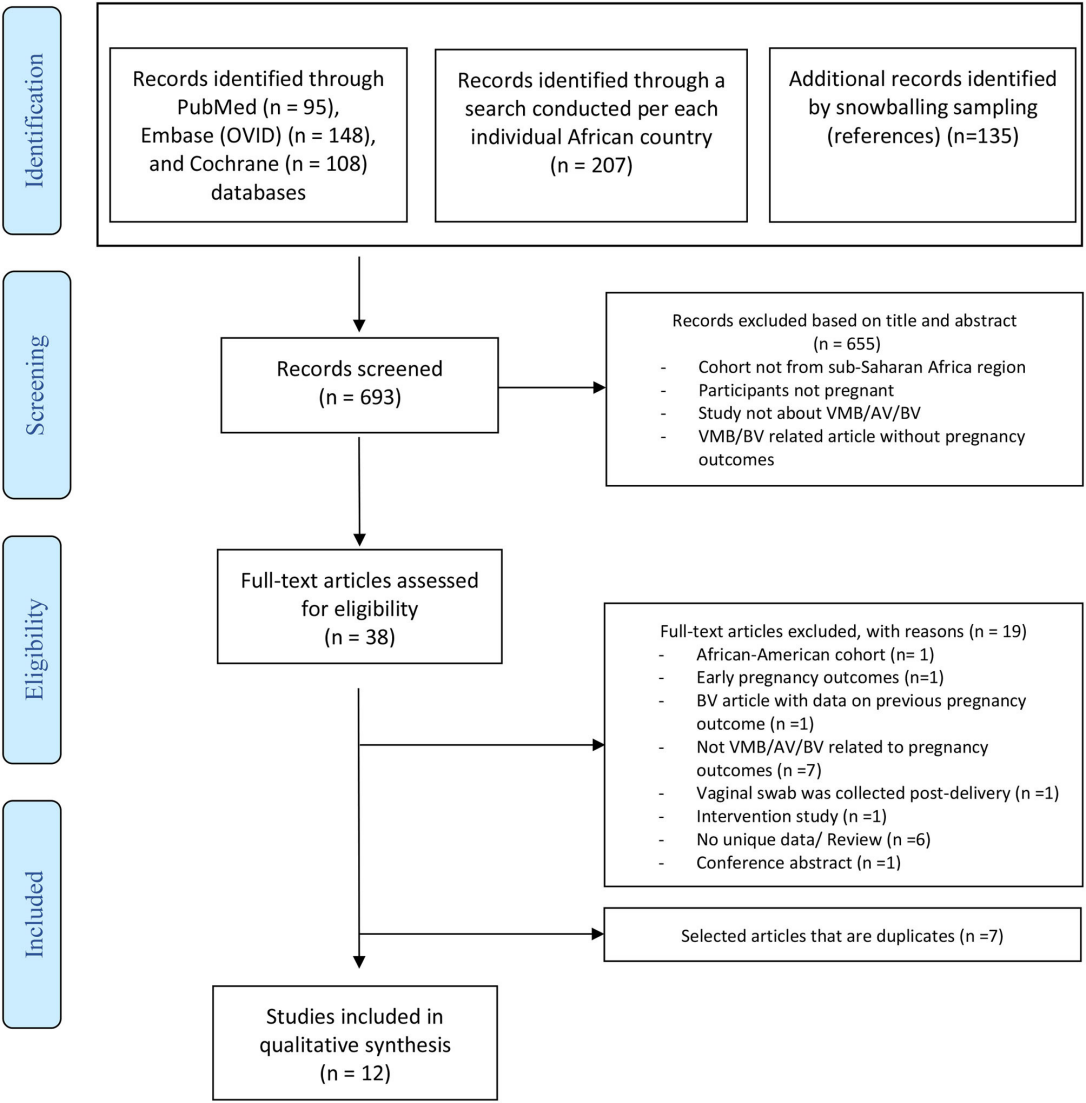
PRISMA based Flow diagram displaying the study selection (69).

The search strategies used yielded 693 records; among them, 12 studies met the inclusion criteria. The study selection process is shown in the PRISMA flow diagram in Figure 1. Of these selected studies, 1 reported the VMB composition and 11 BV status in relation to pregnancy outcomes in pregnant sub-Saharan African women. No retrieved study investigated the role of AV in late adverse pregnancy outcomes in women living in sub-Saharan Africa countries. Seven selected articles were retrieved both via PubMed and Embase (Ovid) (70–76), four articles were retrieved solely via PubMed (31, 77–79), and a conference abstract retrieved on Embase (Ovid) led to an additional article (80). The bibliography from one systematic review retrieved from Cochrane was also assessed (81). However, all the articles that were possibly eligible from it were duplicates on the PubMed or Embase search. Only one article was written in French; the rest were in English. To avoid duplication, the study by Steyn et al. was not included because their results were further analyzed in an already included study (78, 82). The cohorts of women of both studies were the same, and the main conclusions were in line; however, the number of included participants in each analysis was slightly different (78, 82).

### Population and Study Characteristics

**Figure 2:**
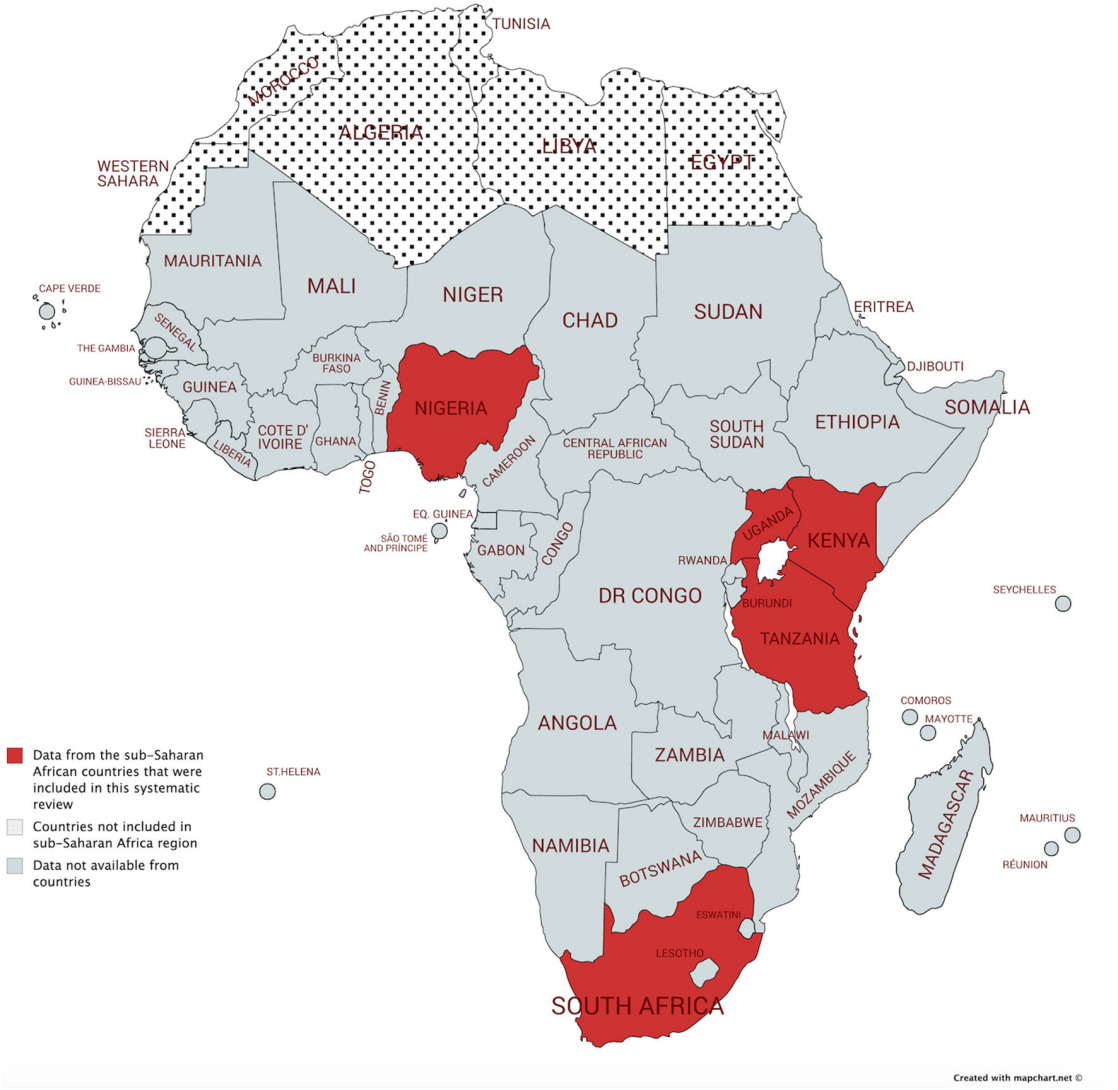
Map of Africa depicting sub-Saharan countries (in gray) where the included studies were conducted (in red) and countries not considered within the sub-Saharan region (spotted). Map created with Mapchart (83).

In total, study reports from five sub-Saharan countries were retrieved (Figure 2) (83). Four cohorts of pregnant women were based in South Africa, three in Nigeria, two in Tanzania and Kenya, and one in Uganda. Tables 1, 2 provide a summary of the cohort characteristics and methodological features of the retrieved studies on the VMB and BV, respectively. All but one study reported baseline characteristics of the women at the time of sampling (84). The GA at sampling was not reported in 9 of 12 studies. Two studies collected their vaginal samples between 13 and 28 weeks of GA (second trimester), and Slyker et al. collected the vaginal samples after 28 weeks of GA (third trimester) (72, 75, 79). Odendaal et al. reported that they collected the vaginal samples soon after enrollment, and Schoeman et al. mentioned in their method that a posterior fornix smear was taken at each visit between 14 and 34 weeks of GA (76, 78).

**Table 1:** Study characteristics and findings of the association between vaginal microbiota (VMB) composition and pregnancy outcomes in pregnant women in the sub-Saharan African region.

**References**	**Country**	**Study design**	**Study population**	**Methods**	**Number of participants**	**Maternal age (years)**	**Gestational age at sampling (weeks)**	**Gravidity/parity**	**Pregnancy outcomes**	**Other genital infections (not BV)**	**Number of women infected with HIV**	**Factors considered when measuring the association**	**Main findings**
Donders et al. (84)	South Africa	Prospective cohort study	Pregnant women at their first antenatal visit	Exocervical scraping and Schröder's classification for *Lactobacillus* morphotypes	256	NR	NR	NR	17/165 neonates with LBW (≤2 kg)	- Candida 25% - CT Ag: 9% - GC: 3%/ - TP: 9% - TV: 36%	0	No factors considered when measuring the association (univariate analysis)	15.7% (*n* = 165) of mothers with grade III Lactobacillus flora at the first antenatal examination gave birth to <2 kg birth weight. This was significantly more often than the 4.7% of mothers with grade I + II *Lactobacillus* flora at the first antenatal examination that gave birth to a birth to <2 kg baby. OR = 3.6 (95% CI, 1.3–11.6, *P* = 0.03)^a^

*BV, bacterial vaginosis; CT Ag, Chlamydia trachomatis antigen; GC, cultured gonococci; NR, not reported; OR, odds ratio; TP, Treponema Pallidum; TV, Trichomonas vaginalis*.

a *Calculated based on the number given in the original paper (83)*.

*Grade I Lactobacillus flora: normal appearance of numerous Lactobacillus morphotypes (84). Grade II Lactobacillus flora: the presence of a diminished amount mixed with other bacteria (84). Grade III Lactobacillus flora: Absence of lactobacilli or overwhelming presence of other bacteria (84)*.

**Table 2:** Study characteristics of studies that investigated the association between BV and pregnancy outcomes of pregnant women in the sub-Saharan African region.

**References**	**Country**	**Study design**	**Study population**	**Follow-up**	**Methods to detect BV**	**Number of participants**		**Maternal age (years)**	**GA at sampling (weeks)**	**Gravidity/parity**	**Other genital infections (*n*)**	**Women infected with HIV**	**Confounding factors considered**
						**BV+**	**BV–**						
Govender et al. (74)	Durban, South Africa	Prospective cohort study	Pregnant women on first visit to ANC (<30 wks) with no vaginal discharge	Followed until delivery	Nugent score	88	80	24 (16–44)	NR	Gr: NR Parity: 3 (0–6)	–Candida: 16 – CT: 14 NG: 5 TP: 20 - TV: 35	9 (5%)	None
Odendaal et al. (76)	Western Cape, South Africa	Randomized control trial	Pregnant women (15–26 weeks) who had had a previous preterm labor or mid-trimester miscarriage. Gr = 1: 464 Gr ≥ 2: 491**	Patients were followed up for the rest of the pregnancy, labor, and puerperium	Gram stain, wet mount smear, amine test, Spiegel test. 3 of 5 Amstel criteria	277 – Gr = 1: 150 – Gr ≥ 2: 127	678 – Gr = 1: 314 – Gr ≥ 2: 364	BV+ group: – Gr = 1 treated: 21.2 ± 5.0 – Gr = 1 placebo: 22.3 ± 4.9 – Gr ≥ 2 treated: 27.1 ± 4.5 – Gr ≥ 2 placebo: 27.9 ± 5.4 BV- group: Gr = 1: 21.3 ± 5.2 - Gr ≥ 2: 28.4 ± 5.4	“Soon after enrollment”: BV + group: –Gr = 1 treated: 20.2 ± 3.1 – Gr = 1 placebo: 20.4 ± 3.0 – Gr ≥ 2 treated: 27.1 ± 4.5 Gr ≥ 2 placebo: 27.9 ± 5.4 BV- group: - Gr = 1: 20.2 ± 2.8 - Gr ≥ 2: 19.7 ± 3.0	Gr (*n*): –Primigravidae: 464 – Multigravidae: 491 Parity: NR	NR	NR	None
Schoeman et al. (78)	South Africa	Double-blind randomized placebo-controlled trial Intervention: Vitamin C	Pregnant women (14–26 weeks) with a history of a previous mid-trimester miscarriage or a preterm delivery	Until delivery	Nugent score	82	116	Vitamin C group: 28 (18–44) Placebo group: 28 (19–45)	NR (samples were taken until 34 weeks)	Gr:–Vitamin C group: 3 (2–8) – Placebo group:3 (2–7) Parity: – Vitamin C group: 1 (0–6) - Placebo group: 2 (0–4)	NR	NR	None
Watson-Jones et al. (75)	Mwanza, United Republic of Tanzania	Prospective cohort study	Women attending antenatal clinic	Followed up to the point of delivery	Nugent's score	459	1,077	23.8 (Mean)	25.4 ± 6.1	Gr (*n*): 1–2: 828 3–5: 566 ≥ 6: 142 Parity: NR	–*Candida*: 454 – CT: 114 NG: 33 - TV: 315	177 (11.7%)	*
Shayo et al. (73)	Mwanza, Tanzania	Cross-sectional descriptive study	Delivering women	No follow-up	Nugent's score	81	202	26 (Median)	NR	Gr: NR Parity: 2 (Median)	*Candida*: 74	15 (5.3%)	None
Slyker et al. (79)	Nairobi, Kenya	Retrospective cohort study	HIV-infected women with ≥28 weeks	Follow-up until 1 year after delivery	Nugent score	144	241	25 (22–28)	32 weeks	Gr: NR Parity: 1 (1–2)	–*Candida*: 124 – CT: 16 NG:8 TP: 10 TV: 65 Any STI: 88	All women were HIV infected	Univariate analysis and stepwise logistic regression
Nakubulwa et al. (70)	Uganda	Unmatched case–control study	Cases: women with confirmed PROM, ≥28 weeks. Controls: Women without PROM in latent phase of labor	No follow-up	Gram stain	2	172	Cases: Age: (*n*) <20: 10 20–35: 73 ≥35: 4 Controls: Age: (*n*) <20: 9 20–35: 69 ≥35: 9	NR	Gr cases (*n*): 1: 31 2–4: 47 >5: 9 Gr controls (*n*): 1:31 2–4: 48 > 5: 8 Parity: NR	–*Candida*: 61 – CT: 6 – NG: 1 – TP: 0 TV: 25 -HSV-Ab: 95 -HSV-ELISA: 51	24 (13.8%)	No further analysis was done for BV due to few numbers
Afolabi et al. (77)	Lagos State, Nigeria	Prospective observational study	Healthy pregnant women (14–36 weeks)	Until 1 week after delivery	Nugent score	64	182	30.9 (20–44) [Mean (range)]	NR	Gr: NR Parity: (*n*) 1: 92 ≥2: 154	NR	0	None
Aderoba et al. (80)	Benin City, Nigeria	Unmatched case–control study	Cases: women with PTL (28–37 weeks). Controls: women with term labor (≥37 weeks)	No follow-up	Nugent score	22	60	Cases: 29.2 ± 5.7 Controls: 29.7 ± 3.8	NR	Gr: NR Parity: 1.89 (0–5) [Mean(range)]^a^	NR	NR	Marital status
Aduloju et al. (72)	Ado-Ekiti, Nigeria	Descriptive cross-sectional study	Pregnant women with abnormal vaginal discharge	Until delivery	Spiegel's method, 3 of 4 Amsel criteria, Nugent score	60	302	BV+: 26.24 ± 6.14 BV–: 28.13 ± 4.38	BV+: 25.27 ± 3.42 BV–: 26.63 ± 2.52	Gr: NR Parity: BV+: 2.7 ± 2.3 BV–: 3.0 ± 1.1	NR	NR	None
Warr et al. (71)	Nyanza region, Kenya	Nested longitudinal cohort study	Pregnant women (HIV uninfected)	9 months postpartum	Nugent score	271	950	22 (19–27)	NR	Gr: 2 (1–4) Parity: 2 (1–3)	–*Candida*: 302 – CT: 65 – NG: 29 – TP: 10 – TV: 79	0	None

*Data are reported as means ± SD, median (IQR), median (range), or n (%), unless otherwise specified*.

*Ab, antibody; ANC, antenatal care; ANC, antenatal clinic; BV, bacterial vaginosis; BW, birth weight; CT, Chlamydia trachomatis; EGA, estimate gestational age; GA, gestational age; Gr, gravidity; HIV, human immunodeficiency virus; HSV-2, human simplex virus type 2; IQR, interquartile range; IUFD, intrauterine fetal death; IUGR, intrauterine growth restriction; IUI, intrauterine infection; LBW, low birth weight; NG, Neisseria gonorrhoeae; NND, neonatal death; NR, not reported; PND, perinatal death; PROM, premature rupture of the membranes; PTB, preterm birth; PTL, preterm labor; SD, standard deviation; SGA, small for gestational age; STI, sexual transmitted infection; TP, Treponema pallidum*.

* *Stillbirth: adjusted for age, height, gravidity, history of stillbirth, HIV at delivery, and maternal anemia. Prematurity: adjusted for age, occupation, gravidity, HIV at delivery, maternal malaria. LBW: adjusted for age, tribe, occupation, height, gravidity, Chlamydia trachomatis at recruitment, HIV at delivery and maternal malaria. IUGR: adjusted for age, tribe, occupation, height, gravidity, baby's sex, maternal malaria and HIV at delivery*.

** *Analysis was done for primigravidae and multigravidae separately*.

All studies used cervicovaginal swabs for sampling (84), except that of Donders et al., which used exocervical scraping swabs. Odendaal et al. obtained posterior vaginal fornix smear from an Ayre spatula, while the other nine studies used vaginal swabs from the anterior, lateral vaginal wall, or posterior vaginal fornix (76). All of the 10 studies used Gram staining, but seven combined it with Nugent score, while two studies used solely Gram staining. The study by Aduloju et al. used three different methods, namely, Spiegel method, Nugent score, and three of the four criteria recommended by Amsel (Table 2) (58–60, 72). Schröder's classification was used by Donders et al. to classify the *Lactobacillus* morphotypes (Table 1) (84).

### Vaginal Microbiota in Relation to Pregnancy Outcomes

The present systematic search identified only one study by Donders et al. that reported the association between VMB diversity and LBW in sub-Saharan African women (84). In this study, the authors reported a significant association between VMB characteristics and newborn's LBW (Table 1). Specifically, pregnant women with a VMB without lactobacilli or with overgrowth of other bacteria (15.7% with grade III *Lactobacillus* flora) at the first antenatal examination had a 3.6 times higher risk (95% CI, 1.3–11.6; *P* = 0.03) of giving birth to a newborn weighting <2 kg compared to those with a *Lactobacillus*-dominated VMB or mixed bacteria with a low abundance of *Lactobacillus* species (4.8% with grades I and II *Lactobacillus* flora) (84).

### Vaginal Conditions in Relation to Pregnancy Outcomes

The included studies reported seven main types of late adverse pregnancy outcomes, namely, LBW, IUGR, SGA, PTB, PROM, pregnancy loss, and intrauterine infection (Table 3).

**Table 3:** The association between bacterial vaginosis infection and adverse pregnancy outcomes in pregnant women living in sub-Saharan Africa.

**Author, year, country**	**Pregnancy outcome (definition)**	***n* or % of women with adeverse pregnancy outcomes over total women tested**	**Number of women with BV-status and adverse pregnancy outcome**		**Odds ratio (95% CI)**		***P*-value**
**Low birth weight**							
Govender et al. (74) South Africa	LBW (<2.5 kg)	36/168	19 BV+	17 BV–	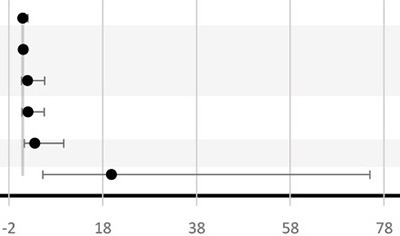	OR = 1.02 (0.5–2.1)^a^	*P* = 0.96^a^
Watson-Jones et al. (75) United Republic of Tanzania	LBW (<2.5 kg)	103/1,260	BV treated NR			OR = 1.08 (0.7–1.8)	NR
			BV untreated NR			OR = 2.02 (0.7-5.7)	NR
Slyker et al. (79) Kenya	6% of 332 infants had LBW (<2.5 kg)	20/332	In total 144 BV+ and 241 BV- women were diagnosed. Cases of LBW were NR per BV+ or BV– group			OR = 2.1 (0.8–5.6)	*P* = 0.10
Afolabi et al. (77) Nigeria	LBW (<2.5 kg)	17/246^a^	9 BV+	8 BV–		**OR** **=** **3.56 (1.3–9.7)**^a^	***P*** **=** **0.01**^a^
Aduloju et al. (72) Nigeria	LBW (not defined)	13/362^a^	10 BV+	3 BV–		**OR** **=** **19.93 (5.3–75)**^a^	***P*** **<** **0.01**^a^
**Intrauterine growth retardation or small for gestational age**							
Watson-Jones et al. (75) United Republic of Tanzania	IUGR (LBW infant born ≥ 37 GA weeks)	4% of 1,117	BV treated NR		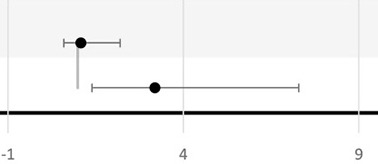	OR =1.09 (0.6–2.2)	*P* = 0.79
Slyker et al. (79) Kenya	SGA (using Mikolajczyk [23] and using Dubowitz-estimated gestational age)	28/311	In total 144 BV+ and 241 BV– women were diagnosed			**OR** **=** **3.2 (1.4–7.3)**	***P*** **<** **0.01**
**Preterm birth**							
Govender et al. (74) South Africa	PTD (<37 GA weeks)	35/268	24 BV+	11 BV–	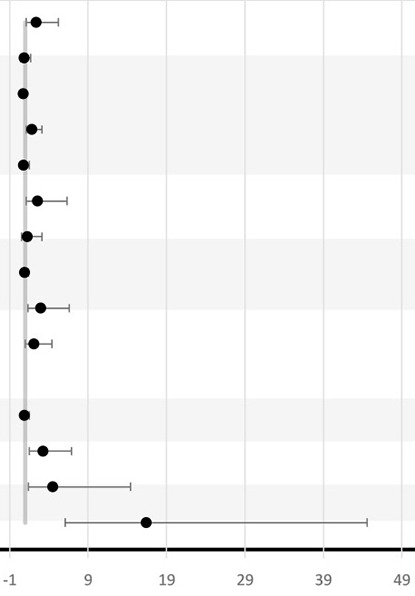	**OR** **=** **2.35 (1.1–5.2)**^a^	***P*** **=** **0.03**^a^
Odendaal et al. (76) South Africa	PTD (<37 GA weeks) treated primigravida	76/377	12 BV+	64 BV–		OR = 0.86 (0.4–1.7)^a^	*P* = 0.66^a^
	PTD (<37 GA weeks) placebo primigravida	77/393	13 BV+	64 BV–		OR = 0.73 (0.38–1.4)^a^	*P* = 0.34^a^
	PTD (<37 GA weeks) treated multigravida	132/421	30 BV+	102 BV–		**OR** **=** **1.83 (1.1–3.1)**^a^	***P*** **=** **0.02**^a^
	PTD (<37 GA weeks) placebo multigravida	114/402	12 BV+	102 BV–		OR = 0.75 (0.4–1.5)^a^	*P* = 0.41^a^
Schoeman et al. (78) South Africa	PTD (<37 GA weeks) tested <20 GA weeks	46/103	23 BV+	23 BV–		**OR** **=** **2.56 (1.05–6.32)**	***P*** **<** **0.03**
	PTD (<37 GA weeks) tested >20 GA weeks	37/95	19 BV+	18 BV–		OR = 1.25 (0.5–3.11)	*P* = 0.59
Watson-Jones et al. (75) United Republic of Tanzania	Prematurity (<37 GA weeks)	12 % of 1,536	BV treated NR			OR = 0.91 (0.6–1.4)	NR
			BV untreated NR			**OR** **=** **2.95 (1.3–6.6)**	NR
Slyker et al. (79) Kenya	9.9% of infants were born preterm (<37 GA weeks)	65/465	In total 144 BV+ and 241 BV–. Cases of LBW per BV+ or BV– group were NR			OR = 2.1 (0.97–4.4)	*P* = 0.06
Shayo et al. (73) United Republic of Tanzania	GA < 37 GA weeks	104/291	29 BV+	67 BV–		OR = 0.88 (0.52–1.53)	*P* = 0.67^a^
Afolabi et al. (77) Nigeria	PTD (<37 GA weeks)	33/246^a^	16 BV+	17 BV–		**OR** **=** **3.24 (1.5–6.9)**^a^	***P<*** **0.01**^a^
Aderoba et al. (80) Nigeria	PTB (<37 GA weeks)	41 PTB vs. 41 term birth	17 BV+	24 BV–		**OR** **=** 4.5 (1.4–14.4)**	***P*** **<** 0.01**
Aduloju et al. 2019 (72) Nigeria	Prematurity (not defined)	21/362^a^	15 BV+	6 BV-		**OR** **=** **16.44 (6.1-44.6)**^a^	***P*** **<** **0.01**^a^
**Preterm rupture of membranes**							
Govender et al. (74) South Africa	PROM (spontaneous rupture of membranes prior to the onset of labor irrespective of the gestation)	24/168	17 BV+	7 BV–	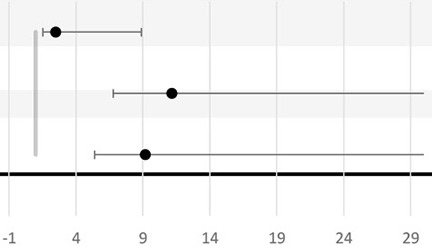	OR = 2.50 (0.98–6.4)^a^	*P* = 0.06^a^
Nakubulwa et al. (70) Uganda	PROM (women in latent labor with clear fluid from perineum and through sterile speculum examination)	87 PROM cases vs. 87 controls (without PROM)	0 BV+	2 BV–		No analysis conducted due to few numbers	NR
Afolabi et al. (77) Nigeria	PROM (not defined)	27/246^a^	19 BV+	8 BV–		**OR** **=** **9.18 (3.8–22.3)**^a^	***P*** **<** **0.01**^a^
Aduloju et al. (72) Nigeria	PROM (not defined)	22/362^a^	14 BV+	8 BV–		**OR** **=** **11.18 (4.4–28.1)**^a^	***P*** **<** **0.01**^a^
**Pregnancy loss**							
Govender et al. (74) South Africa	Pregnancy losses (miscarriage, stillbirths, neonatal deaths)	25/168	14 BV+	11 BV–	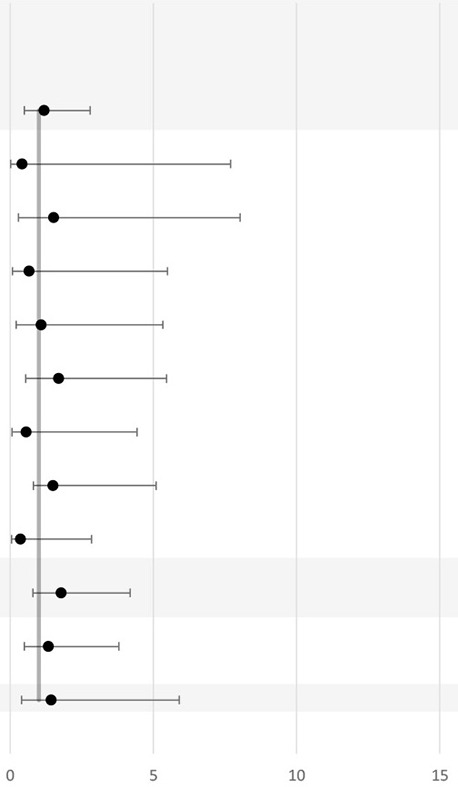	OR = 1.19 (0.5–2.8)^a^	*P* = 0.69^a^
Odendaal et al. (76) South Africa	IUD (not defined) under primigravidae treated group	5/377	0 BV+	5 BV–		OR = 0.42 (0.02–7.7)^a^	*P* = 0.55^a^
	IUD (not defined) under primigravidae placebo group	7/393	2 BV+	5 BV–		OR = 1.53 (0.3–8.0)^a^	*P* = 0.62^a^
	PND (not defined) under primigravidae treated group	8/377	1 BV+	7 BV–		OR = 0.67 (0.08–5.5)^a^	*P* = 0.71^a^
	PND (not defined) under primigravidae placebo group	9/393	2 BV+	7 BV–		OR = 1.08 (0.2–5.3)^a^	*P* = 0.92^a^
	IUD (not defined) under multigravidae treated group	16/421	4 BV+	12 BV-		OR = 1.71 (0.5-5.5)^a^	*P* = 0.36^a^
	IUD (not defined) under multigravidae placebo group	13/402	1 BV+	12 BV–		OR = 0.57 (0.1–4.4)^a^	*P* = 0.59^a^
	PND (not defined) under multigravidae treated group	25/421	7 BV+	18 BV–		OR = 2.06 (0.8–5.1)^a^	*P* = 0.12^a^
	PND (not defined) under multiigravidae placebo group	19/402	1 BV+	18 BV–		OR = 0.37 (0.1–2.8)^a^	*P* = 0.34^a^
Watson-Jones et al. (75) United Republic of Tanzania	Stillbirth (≥ 22 GA weeks)	42/1,536 (2.7%) women experienced a stillbirth or IUFD	Treated BV NR			OR = 1.79 (0.8–4.2)	*P* = 0.18
Warr et al. (71) Kenya	Stillbirth (fetal death at ≥20 weeks gestation and included births without signs of life)	19/1,221	5 BV+	14 BV–		OR = 1.34 (0.47–3.80)	*P* = 0.58
Afolabi et al. (77) Nigeria	Pregnancy loss (14–28 GA weeks)	9/246^a^	3 BV+	6 BV–		OR = 1.44 (0.4–5.9)^a^	*P* = 0.61^a^
**Intra-uterine infections**							
Govender et al. (74) South Africa	Intrauterine infection (diagnosed on clinical findings of maternal pyrexia and tachycardia, fetal tachycardia, uterine tenderness, and/or offensive liquor)	15/168	11 BV +	4 BV–	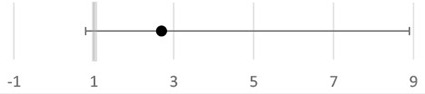	OR = 2.71 (0.8–8.9)^a^	*P* = 0.09^a^

*Forest plot of the association between bacterial vaginosis (BV) infection and adverse pregnancy outcomes in pregnant women living in sub-Saharan Africa. P-values indicate differences in outcomes between pregnant women with BV (BV+) and without BV (BV–) in individual studies (statistically significant differences in bold = P < 0.05). Data are expressed as odd's ratio's (OR). OR = 1 is indicated as a gray line in the forest plots. Adjusted OR (ªOR) were calculated based on the values given in the selected articles as described by Altman or Pagano and Gauvreau, and P-value based on Sheskin (59–61)*.

** *Adjusted odds ratio for marital status*.

a *Calculated based on the number given in the original paper*.

*BV, bacterial vaginosis; CI, confidence interval; IUD, intrauterine death; IUGR, intrauterine growth restriction; LBW, low birth weight; NND, neonatal death; OR, odds ratio; PND, perinatal death; PTB, preterm birth; PTD, preterm delivery; PTL, preterm labor; PROM, premature rupture of membrane; SGA, small for gestational age*.

Six studies looked at the association between BV and LBW (72, 74–77, 79). All studies, except that of Odendaal et al., used the traditional cutoff value of birthweight <2.5 kg to define LBW and to calculate the association with BV positivity (76). Independent studies from South Africa, the United Republic of Tanzania, and Kenya did not find any statistical association between BV and LBW (74, 79, 85). However, both studies from Nigeria had independently reported a positive association between BV and LBW with OR ranging from 3.56 to 19.93 (72, 77). The precision of the OR in the latest study is low since the 95% CI is broad (95% CI, 5.3–75) (72). In Odendaal et al., the difference in mean birthweight between BV-positive and BV-negative groups of South African pregnant women was used as the outcome, and the association was stratified by gravidity and treatment (metronidazole or vitamin C) (76). There was a significant difference in mean birth weights among the metronidazole-treated group (2.48 kg), the placebo-treated group (2.76 kg), and BV-negative group (2.75 kg) (*P* < 0.01) (76). This effect was confined for multigravid women but not for primigravid women. Only one study from the United Republic of Tanzania studied IUGR as an outcome and found no significant difference in IUGR prevalence between the BV-treated women and the BV-negative women (75). One study conducted in Kenya reported a 3.2-fold increase in the incidence of SGA in BV-positive women compared to BV-negative women (95% CI, 1.4–7.3; *P* = 0.005) (79).

Nine studies investigated the association between BV and PTB (72–80) (Table 3). In total, seven studies reported a significant positive association between BV-positive individuals and PTB (72, 74–78, 80). However, the difference in PTB incidence was not consistent across all subgroups (Table 3). For example, Shoeman et al. reported a significant increase in incidence (OR, 2.56; 95% CI, 1.05–6.32; *P* < 0.03) of PTB attributable to BV only in the subgroup of women tested for BV before 20 weeks of GA (78). In addition, Odendaal et al. reported a significant increase (OR, 1.83; 95% CI, 1.1–3.1) in incidence of PTB only among the metronidazole-treated subgroup and not in the placebo-treated subgroup compared to the BV-negative multigravid women (76). Furthermore, Watson-Jones et al. observed that Tanzanian women who received treatment in their group were less likely to have a PTB compared to women who were BV negative (OR = 0.91; 95% CI, 0.6–1.4) (75). Even though the study conducted by Watson-Jones et al. did not report a separate *P*-value, the OR for PTB when comparing treated BV-positive individuals to untreated BV-positive individuals was higher than 1 (OR = 2.95; 95% CI, 1.3–6.6). Moreover, neither the study conducted by Slyker et al. in Kenya nor the study by Shayo et al. in the Republic of Tanzania observed a significant association between BV-positive individuals and PTB in their study cohorts (73, 79). In Nigeria, three studies reported a significant positive association between BV-positive individuals and PTB with a wide range (3.24–16.44-fold) increase in incidence of PTB in BV-positive women compared to BV-negative women (72, 77, 80).

Two of these three studies found a significant increase in incidence of PROM: 9.18-fold (95% CI, 3.8–22.3) and 11.18-fold (95% CI, 4.4–28.1), respectively, in BV-positive women compared to BV-negative women (72, 77), while Govender et al. found a 2.5-fold (but not significant) increase in incidence (95% CI, 0.98–6.4) of having PROM associated with BV positivity (74). That study also reported an association between BV and intrauterine infection in the sub-Saharan region (OR = 2.71; 95% CI, 0.8–8.9) (74).

Due to the paucity of data, late miscarriage, stillbirth, IUD, perinatal death, and neonatal death outcomes from the included studies were all considered as different pregnancy loss measures in this review (Table 3). None of the studies included reported a significant association between BV and pregnancy loss. Only Govender et al. investigated the association between BV and intrauterine infection in the sub-Saharan region (OR = 2.71; 95% CI, 0.8–8.9) (74).

### Risk of Bias Within Studies

Five studies had a low risk of bias, six had moderate risks of bias, and one had high risk of bias (Table 4). One of the first studies that aimed to assess the vaginal microbiota of African women was from Donders et al. This study has a high risk of performance bias mainly because it used outdated culture techniques and failed to mention patient's characteristics and which kind of strategies they used in order to reduce confounding (84). Selection bias and measurement bias factors such as age, parity, smoking status, obstetric history, and gestational age of sampling were not reported (84, 86). All cohort studies included are at risk for selection bias as described by the JBI critical appraisal tool. None of them addressed “the numbers of loss to follow up” or “which strategies were used to address the potential loss to follow up” (66). Selection bias could have also played a role in the case–control study conducted by Nakubulwa et al. mainly because cases were not matched to controls (70). Both randomized control trials (RCTs) included were low for risk of bias (76, 78).

**Table 4:** Assessment of risk of bias according to the Joanna Briggs Institute Critical Appraisal Tool.

**Study**	**Q1**	**Q2**	**Q3**	**Q4**	**Q5**	**Q6**	**Q7**	**Q8**	**Q9**	**Q10**	**Q11**	**Q12**	**Q13**	**% yes**	**Risk^***b***^**
**Cohort studies**															
Warr et al. (71)	✓	✓	✓	×	×	✓	×	✓	×	×	✓	N/A	N/A	55% (6/11)	Moderate
Watson-Jones et al. (75)	✓	✓	✓	✓	✓	✓	✓	✓	×	×	✓	N/A	N/A	82% (9/11)	Low
Afolabi et al. (77)	✓	✓	✓	×	✓	✓	?	✓	×	×	✓	N/A	N/A	68% 7.5/11	Moderate
Govender et al. (74)	✓	✓	✓	×	×	✓	?	✓	×	×	✓	N/A	N/A	59% 6.5/11	Moderate
Slyker et al. (79)	✓	✓	✓	✓	✓	✓	×	✓	×	×	✓	N/A	N/A	73% 8/11	Low
Donders et al. (84)	×	✓	×	×	×	N/A	?		×	×	✓	N/A	N/A	25% 2.5/10	High
**Cross sectional studies**															
Aduloju et al. (72)	✓	✓	✓	✓	×		?	✓	N/A	N/A	N/A	N/A	N/A	69% (5.5/8)	Moderate
Shayo et al. (73)	✓	✓	✓	✓	×	×	?	✓	N/A	N/A	N/A	N/A	N/A	69% (5.5/8)	Moderate
**Case-control studies**															
Aderoba et al. (80)	×	×	✓	✓	✓	✓	✓	✓	✓	✓	N/A	N/A	N/A	80% (8/10)	Low
Nakubulwa et al. (70)			✓	✓	✓	×	×	×	✓	✓	N/A	N/A	N/A	50% (5/10)	Moderate
**Randomized controlled trial**															
Schoeman et al. (78)	✓	✓	✓	✓	✓	?	✓	✓	?	✓	?	✓	✓	84% 11/13	Low
Odendaal et al. (76)	✓	✓	✓	?	?	?	✓	×	?	✓	✓	✓	✓	77% 10/13	Low

*Studies included in this review were assessed for risk of bias according to JBI (Joanna Briggs Institute) Critical Appraisal Tool, detailed in reference (66) and Supplementary Material 2. ✓, Indicates yes (1 point); ×, Indicates No (0 points); “?”, Indicates unclear (0.5 points). Risk^b^: The risk of bias was ranked as high when the study score reached up to 49%, moderate when the study score reached from 50 to 69%, and low when the study score reached more than 70%. N/A, not applicable*.

## Discussion

The aim of this study was to review evidence on the association between the diversity of the vaginal bacterial ecosystems in sub-Saharan African women and pregnancy outcomes. To identify all relevant work on the topic, studies on the association between VMB diversity, presence of BV and AV, and pregnancy outcomes were systematically identified and reviewed.

### VMB Characteristics and Pregnancy Outcomes

Several studies in North America have investigated the association between VMB characteristics and PTB. DiGiulio et al. observed that an increased prevalence of diverse VMB is associated with PTB (51). In a relatively small number of participants, they also found that a high abundance of *Gardnerella* or *Ureaplasma* with a low abundance of *Lactobacillus* species was associated with PTB (51). Stout et al. observed in a predominantly African-American cohort that PTB was associated with a significant decrease in VMB richness and diversity, rather than changes in specific taxa or a taxon (43). In women who delivered preterm, they did observe that *Ureaplasma* abundance increased in the second and third trimesters; however, this increase was not significant (43).

On the other hand, the longitudinal study conducted by Romero et al. did not find an association between PTB and any abundance of specific taxa or vaginal ecosystem characteristic (52). These differences between studies might be attributed to the frequency and timing of sample collection, ethnical differences in the study populations, and methods used to detect the microbial species (43). Nevertheless, these combined results raise further interest in investigating the relationship between VMB characteristics and PTB, LBW, and other late adverse pregnancy outcomes in SSA women.

To date, only the study by Donders et al. has investigated the association between VMB diversity and pregnancy outcomes in sub-Saharan Africa (84). This work suggested an association between VMB with no or low lactobacilli and risk of having a child weighting <2 kg (84). Very low birthweight can be an indicator of PTB (84). As reported by two independent non-pregnant cohort studies from sub-Saharan Africa, a correlation between the presence of non-*Lactobacillus* dominant VMB and increased inflammatory cytokines and chemokines in the vagina has been observed (14, 87, 88). A longitudinal study in the United States of America that consisted of pregnant women predominantly of African ancestry observed that proinflammatory cytokines in the vaginal fluid were correlated with taxa associated with dysbiosis and PTB (89). More recent studies suggest that high production of proinflammatory cytokines due to VMB dysbiosis contributes to further activation of the host inflammatory response in the reproductive tract, which in turn can induce labor, also a premature one (14, 87, 89, 90).

The work by Donders et al. is also one of the first that studied the genital bacteria in pregnancy in sub-Saharan Africa (84). The culture-based approach and analysis methods (Schröder's classification for lactobacilli morphology detection) used are now outdated and poorly described in the paper (84). The main limitations of using culture-dependent methods are that only medium-specific species can grow and that highly abundant or fast-growing VMB species will limit the detection of others (48, 91, 92). In the last decade, the use of culture-independent, molecular techniques, such as PCR-based techniques and sequencing of the 16S ribosomal (r)RNA gene allow for an unprecedented high-resolution detection of the microbiota (93). Hence, more studies using molecular microbiology techniques are needed to confirm the findings by Donders et al. and to further investigate whether the VMB also has a role in other adverse pregnancy outcomes, such as PTB or neonatal mortality, which remain burdensome in sub-Sahara Africa.

### The Association Between BV and Adverse Pregnancy Outcomes

Three of the six studies included found a positive association between BV-positive individuals and LBW (72, 76, 77). However, the study by Watson-Jones et al. did report that untreated BV was associated with LBW only before adjustment for age, tribe, occupation, height, gravidity, *Chlamydia trachomatis* infection at recruitment, HIV at delivery, and maternal malaria (75). Furthermore, in three studies that did not observe an association between BV and LBW, there was confounding effect of gravidity or parity reported (75, 76, 79).

The findings in sub-Saharan African women are in contrast with those in a healthy, homogeneous, Caucasian Danish population study (94). In this Danish study, the authors found an association between BV and LBW in nulliparous women (adjusted OR, 4.3; 95% CI, 1.5–12) (94). In the same study, this association was not observed for multiparous women (94).

The effect of BV on fetal and neonatal growth is difficult to interpret, as LBW can result from either IUGR, SGA, or premature delivery. Even though Slyker et al. did not report a significant association between BV and LBW during pregnancy, the newborns of mothers with BV were significantly smaller for their gestational age than those from BV-negative women (79). However, in this study, Dubowitz scoring was used to determine gestational age at birth, which could have influenced the results. This score is feasible to use in low-resource settings and is as reliable a method as ultrasound (the gold standard). However, the Dubowitz score can overestimate the gestational age by 3.9 days (±7.1) compared to ultrasound (95). This may have masked the potential association between BV and PTB (95, 96). The SGA results of Slyker et al. are in line with the results from the Danish population study in which they also observed a significant association between BV and SGA in nulliparous women (adjusted OR, 1.6; 95% CI, 0.7–3.1) (94). These differences in LBW according to gravidity and parity, and the role of BV in this, need to be further investigated.

Although most studies (7 of the 11) reported a significant association between BV-positive status and PTB (72, 74–78), this evidence is weakened by the fact that two of the seven studies that reported a positive association between BV-positive individuals and PTB did not consider the possible confounding effect of baseline characteristics, such as mean maternal age, marital status, and antenatal antibiotic use (74, 77). However, it is unlikely that these variables would cause such an increased prevalence of PTB or other adverse pregnancy outcome in the BV-positive group (97).

On the other hand, the results of the two studies that did not find a significant association between BV and PTB should be analyzed carefully (73, 79).One of these two studies did not make a comparison of the baseline characteristics between BV-positive and BV-negative women; thus, other differences between the two groups could have played a role in masking the association (73). The other study observed a borderline significance between BV and PTB (*P* = 0.06). However, the usage of Dubowitz score in their study to detect the gestational age could have also cause a misclassification of PTB as outcome (79).

Schoeman et al. showed that women who were diagnosed with BV before 20 weeks of GA were significantly more at risk for PTD than women diagnosed after 20 weeks of GA (78). Thus, gestational age at diagnosis should also be taken into account. This is in agreement with the results of a systematic review conducted by Leitich et al., which confirmed the hypothesis that BV early in pregnancy, or in the first 16 weeks of GA, is a much stronger risk factor for PTB than BV late in pregnancy (22). In their meta-analysis, included studies were not restricted to those performed in sub-Saharan Africa (22). Overall, more evidence is needed in African women to confirm this association.

A positive association between BV and PTB was found in several other studies or meta-analyses that were not explicitly limited to the sub-Saharan African population (20, 22, 98). However, in the study conducted by Thorsen et al., BV was not associated with PTB in a Danish population (94). In low-income countries, including some countries from sub-Saharan Africa, spontaneous preterm PROM contributes to a third of all PTB (17, 99). In the current review, two studies found a positive association between BV and PROM (72, 77). Another study could not assess the association since only two women had PROM and were both BV negative (70). As also seen in other populations, such as Northern American women, the association between BV and PROM seems relatively strong (98, 100). PROM and PTB are probably attributable to both inflammation and bacterial enzymes, such as proteases, which are being produced by certain bacteria (98). Both can have a causative role in the disruption of the collagen network of the membrane morphology, enhance placental inflammation, and activate uterine contractions (101). PROM can be caused by intrauterine infection, and one of its main risk factors can be BV (102). In the South African cohort, Govender et al. observed a non-significant 2.7-fold increase in odds in the association between BV and intrauterine infection (74). As reviewed in Redelinghuys et al., increased microbial activity from certain anaerobic Gram-negative bacteria, such as *Gardnerella vaginalis*, particularly when they are heavily colonizing the vagina, may lead to toll-like receptors (TLR) activation. Subsequently, TLR activation induces the production of proinflammatory cytokines and activation of the adaptive immune system in the reproductive tract (103–105). IUD might be induced by proteases that stimulate the production of proinflammatory cytokines, and PROM, chorioamnionitis, and preterm labor might be induced by the increased phospholipase A2 production, also stimulated by proteases (74, 103, 105, 106).

BV is characterized by the absence of inflammation, such as no increase in circulating leukocytes, decreased production of interleukin (IL)-8, elevated levels of IL-1β and IL-6, and absence of clinical signs of inflammation, such as pain or redness of the vaginal mucosa (105, 107, 108). However, it is hypothesized that women with a genetic predisposition for pathological inflammatory responses to BV are more susceptible to having PROM or PTB (105, 109). For instance, BV can activate tumor necrosis factor (TNF)-α expression in women (110). Furthermore, it has been observed that some women with African ancestry living in North America, who have a TNF-α polymorphism (TNF-α−308G>A), have a 6-fold increased risk of preterm labor (108, 111). Other types of polymorphisms that may affect specific cytokine and chemokine secretion, such as IL-6, have also been observed among women with sub-Saharan African ancestry (12, 110). Thus, there seems to be an interaction between the host immune system and the VMB, especially in its dysbiotic state, and this interaction can be enhanced by host genetic factors (14, 105).

Pregnancy loss variables were assessed in 5 out of 12 of the included studies, and none found an association between BV and pregnancy loss (71, 74–77). Conclusions whether there is an association between BV and second trimester pregnancy losses vary between studies from different global regions. In line with the findings of this systematic review, a study conducted in North America also did not find an association between BV and second trimester pregnancy loss (112). However, other studies conducted in England, Belgium, Pakistan, and the United States of America did independently associate BV with second trimester pregnancy loss in their cohorts (40, 113–115). As for third trimester pregnancy loss due to BV, scarce data are available. It is possible that the variation in the association between BV and pregnancy loss might be explained by ethnicity and geographical region, just as the differences in the BV prevalence, especially in women with asymptomatic BV (18). Therefore, more studies are needed to evaluate the correct associations and to explain differences in the association between different populations.

### AV and Pregnancy Outcomes

In 2017, Kamboo and Africa conducted an electronic search on studies investigating the association between AV and pregnancy outcomes (116). Similar to our findings, they reported a paucity of studies on the topic (116). To this date, there is a need for published research on the role of AV in adverse pregnancy outcomes for the sub-Saharan Africa region. Nevertheless, in the past years, there has been growing interest in this topic. Donders et al. found an association between AV and increased early PTB rate (<35 weeks of GA) (OR = 3.2; 95% CI, 1.4–9.1; *P* = 0.04) but not PTB rate (25–37 weeks of GA) in a Belgian population (31). Moreover, in a prospective study conducted in Saudi Arabia, there was an association between AV and PTB (adjusted OR, 3.06; 95% CI, 1.58–5.95; *P* < 0.01) and PROM (adjusted OR, 6.17; 95% CI, 3.24–11.7; *P* < 0.01) but not LBW (adjusted OR, 2.13; 95% CI, 0.85–5.4; *P* = 0.11) (32). They also observed that severe forms of AV significantly increased the incidence of PTB (*P* < 0.01) and PROM (*P* < 0.01) compared to less severe forms of AV (32). As mentioned, the present study did not retrieve any full peer-reviewed article from sub-Saharan Africa. However, via the Embase (Ovid) search engine, a conference abstract that investigated the role between AV and PTB was found (117). Unfortunately, after correspondence with the authors, it became clear that no paper has been published yet on the study. However, this suggests that research about the role of AV in adverse pregnancy outcomes might be ongoing in sub-Saharan Africa (116).

### Factors Influencing the Vaginal Diversity and Pregnancy Outcomes

Among the factors already mentioned, there are many other methodological, biological, or sociological factors that should be considered when analyzing the VMB, vaginal dysbiotic conditions, and their role with pregnancy outcomes.

In the past two decades, the introduction of molecular genotypic methods, such as 16S rRNA-based phylogeny, revealed many other species and the extensive diversity of species within a genus, including the *Lactobacillus* genus (118, 119). It has been increasingly recognized that some species until now classified as part of the *Lactobacillus* genus or other bacterial genera have different phylogenetic, ecological, and physiological characteristics that match more with other bacterial genera (118). Therefore, common genera or species in the reproductive tract, including *Lactobacillus* and *Gardnerella*, are undergoing taxonomic reclassifications based on phylogenetic analyses (118–120).

Although the different taxonomic classification might not directly impact the clinical community, food and health-related industries, lay-person, and public health approaches, it will assist in forthcoming microbiology and biomedical research (118). Reclassifications of different taxa based on phylogenetic genera that include species containing the same ecological and metabolic properties, and the introduction of separate species, might provide new and more specific insights on their beneficial or pathogenic potential. In turn, when species result is clinically relevant, this information might be useful for diagnostics or therapeutic approaches beneficial for female reproductive and maternal health.

Furthermore, co-occurring infections with various organisms, such as *Candida albicans, Trichomonas vaginalis*, and other sexually transmitted infections, are burdensome in sub-Sahara Africa and can induce inflammatory responses in the vagina (69, 121). Therefore, these organisms should be considered potential confounders when investigating the role between vaginal dysbiotic conditions or VMB and adverse pregnancy outcomes. Donders et al. observed that women with absent *Lactobacillus* morphotypes giving birth to a child with LBW (≤ 2 kg) had an average of 1.15 infections with *T. vaginalis, C. trachomatis*, gonococci, or syphilis, against an average of 0.7 infections when the birthweight was >2 kg (84), while women with a *Lactobacillus*-dominated VMB, or mixed bacteria with a low abundance of *Lactobacillus* species, had an average of 0.21 infections, irrespective of the birthweight of their infant (84). The same study also reported a positive association between absent *Lactobacillus* morphotypes and *T. vaginalis, C. trachomatis*, and gonococci (84). However, since the authors did not aim to detect BV in their cohort, they raised the question of whether a VMB with absent *Lactobacillus* morphotypes could be primarily associated with BV, rather than with these detected pathogens (84). This could be especially the case since BV is a dysbiotic vaginal condition, and women with BV frequently have other vaginal infections. Interestingly, only one of the included studies on BV corrected for other co-occurring infections. This raises the question of whether the associations found can be attributed to BV alone, to other specific infections, or a combination of BV and other infections. The study conducted by Govender et al. reported a 2.35-fold higher incidence of having a PTB and a 2.71-fold higher incidence of having an intrauterine infection in women with BV compared to women having other infections or no infection (74). These findings advocate for BV contributing to PTB and intrauterine infection independently of other co-occurring infections.

However, there was no significant increase in OR for the association of BV with LBW, pregnancy loss, and PROM (74). As already mentioned above, the downside of the study of Govender et al. is that no comparison was made for the baseline characteristics between the two groups. Thus, it is unknown whether differences between the baseline variables (such as age, disease, or other sexual risk factors) could influence the outcome and cause chance bias (74).

When comparing populations, besides genetics, different sociocultural factors should also be considered. Those are, for instance, socioeconomic status, poor access to healthcare services, poor nutrition, hygiene and sexual practices, education, and household income, which have been associated with VMB differences even in very homogeneous study groups (122). Moreover, local epidemics of STI and burden of adverse pregnancy outcomes might also explain population differences in VMB composition across sub-Saharan Africa (10, 55, 123–127). Studies across Europe, Latin America, and sub-Saharan Africa have suggested that school attendance and school-based sexual and reproductive health programs promote acceptance of care during pregnancy and avoidance of early high-risk sexual activities that might lead to increase susceptibility to BV or other genital infections (128, 129). Nonetheless, school attendance in certain parts of sub-Saharan Africa is lower than in other world regions due to social (for instance, gender inequality), economical, and infrastructural reasons (129–131); therefore, access to sexual education remains a problem for these populations, particularly girls (129). Even if school education is provided in certain urban communities in sub-Sahara Africa, as in other low- and middle-income countries, misconceptions and stigma about sexual and reproductive-health-related issues persist (132). In addition, inadequate or incorrect knowledge of symptoms of certain diseases might lead to delayed recognition of vaginal dysbiotic conditions in women, delayed diagnosis of possible sequalae, and delayed ante- and perinatal care.

Furthermore, in many resource-constrained settings, laboratory diagnosis, even in the form of Gram staining, is not practical (133), and even if diagnostic services are available, treatment is mostly the result of syndromic management (treatment directed to the organisms that most often cause specific signs and symptoms) (133). This type of management misses asymptomatic BV or vaginal dysbiotic condition, which are most of the cases. Healthcare-seeking behavior of patients, integration of treatment within antenatal care services, knowledge of health providers on the efficacy of chosen drugs, and drugs availability are important when implementing syndromic management in the public health system and are still challenging in certain parts of sub-Saharan Africa (133).

### Limitations

The inclusion of different study designs contributes to the heterogeneity of data included in this systematic review. Most studies had an observational design, and their analysis should be interpreted with caution since causality and confounding are two factors that can lead to the overestimation of an association (OR), and the latter were not always adjusted for. The decision to include intervention and cross-sectional studies and studies that did not adjust for potential confounders was due to the limited number of original studies available on the VMB, AV, and BV in pregnant women in the sub-Saharan region (134–136). Another factor contributing to the heterogeneity of included studies is the different measurements used for the same outcome and the annotation of the GA or trimester at sampling collection. Indeed, the GA was not always reported or not consistently so. Previous longitudinal studies have demonstrated differences in VMB characteristics throughout the pregnancy, even within the same subjects (43, 51). The exact mechanisms behind VMB changes during pregnancy and whether they provide any physiological benefit to either mother or fetus are still topics of debate (14). Even though the most appropriate timing for biospecimen collection during pregnancy is unclear, it remains essential to consider the gestational age of pregnancy at the time of collection when comparing findings from different studies. The limitations of Gram staining and Spiegel and Nugent scoring system should also be taken into account. Even though Nugent scoring is standardized, reproducible, reliable, and its degree of inter- and intraobserver variability is lower than for the Amsel criteria, all three methods require testing facilities, training, and quality control procedures, which were not always stipulated in the included articles (18). Moreover, Gram staining only provides gross morphological-based diagnosis, with only a limited understanding of the VMB composition and relative abundance of bacterial species (18, 137, 138). The use of ORs to report outcomes can lead to overestimation of the risk in case of a positive association, and this overestimation can increase if the outcome is common. As observed in the meta-analysis conducted by Flynn et al., even after using a random effect model, the summary OR of three (LBW, PROM, premature onset of labor) of the four adverse pregnancy outcomes investigated exceeded the relative risk, possibly due to pooling different subset of cohort studies (21).

## Conclusion and Implications for Future Research

An overview of the evidence on the role of VMB dysbiosis status and related vaginal dysbiotic conditions with late adverse pregnancy outcomes is essential to improve maternal and newborn health sub-Saharan Africa. In this systematic review, 12 studies investigated the association between *Lactobacillus*-deficient VMB or BV and seven different types of late adverse pregnancy outcomes. Due to the various methodological differences between the retrieved studies, only the individual strengths of association were summarized per late adverse pregnancy outcome.

Unfortunately, no published evidence was found on the association between AV and late adverse pregnancy outcomes in sub-Saharan Africa, which advocates for research on the topic.

Furthermore, only one study was retrieved to support a positive association between *Lactobacillus*-deficient VMB and LBW. The association between VMB dysbiosis and other types of late adverse pregnancy outcomes could not be assessed. Eleven studies were retrieved that investigated the association between BV and seven late pregnancy outcomes. Two out of four studies independently reported a positive association between BV and PROM. Across the included studies, there were discrepancies to support the association between BV and PTB or LBW. At least two studies conducted in Nigeria supported the association between BV and PROM, PTB, and LBW in Nigerian women. The association between BV and any type of pregnancy loss variable was not been observed in any study. Moreover, there was a single evidence supporting the association between BV and intrauterine infection, BV and SGA, or the absence of association between BV and IUGR, respectively. Thus, more high-quality observational studies are needed to investigate the association between BV and PTB, LBW, PROM, SGA, and intrauterine infection within populations in this geographical region.

Prospective studies of pregnancy complications should correct for baseline characteristics and take gestational age at collection into account. Moreover, other co-occurring infections and use of antibiotic treatments should also be measured and corrected for. From this systematic review, it is difficult to ascertain whether the findings are unique to sub-Sahara Africa or ethnic background and whether adverse pregnancy outcomes attributable to VMB dysbiosis or related conditions are reflected in other geographical regions. Therefore, it is of interest to expand on this research topic with further studies across world regions considering differences, such as VMB characterization methodologies, clinical classification of BV and AV, and other known confounding factors between populations.

The high burden of BV, sexually transmitted infections, and pregnancy complications, and the potential role of the VMB dysbiosis on them, supports the need for future studies to determine the vaginal eubiotic state among sub-Saharan African women adequately. Further investigations on the physiological and pathological function of the VMB and related vaginal dysbiotic conditions, especially AV and BV, during pregnancy will provide evidence for health promotion strategies and the prevention of health conditions among mothers, which will in turn have a meaningful impact on public health.

## Author Contributions

NJ and MS performed the systematic search, screening, inclusion of the study and wrote the introduction, materials, methods, results, and discussion sections. SA-N and SM critically reviewed the manuscript. RP and EA conceived the original idea, supervised the study, and critically reviewed and edited the manuscript. All authors contributed to the final versions of the manuscript.

## Conflict of Interest

The authors declare that the research was conducted in the absence of any commercial or financial relationships that could be construed as a potential conflict of interest.
