# Supplementary material for: The Association Between Vaginal Microbiota Dysbiosis, Bacterial Vaginosis, and Aerobic Vaginitis, and Adverse Pregnancy Outcomes of Women Living in Sub-Saharan Africa: A Systematic Review
Source: Front Public Health. 2020 Dec 10;8:567885. doi: 10.3389/fpubh.2020.567885 (PMC7758254; doi:10.3389/fpubh.2020.567885)
Supplement: Supplementary file 1 [file Table_3.DOCX]

Supplementary Material

Supplementary Table 1. PRISMA checklist displaying the page numbers where the section or topic is provided.

| **Section/topic** | **#** | **Checklist item** | **Reported on page #** |
| --- | --- | --- | --- |
| **Title** | | |  |
| Title | 1 | Identify the report as a systematic review, meta-analysis, or both. | 1 |
| **Abstract** | | |  |
| Structured summary | 2 | Provide a structured summary including, as applicable: background; objectives; data sources; study eligibility criteria, participants, and interventions; study appraisal and synthesis methods; results; limitations; conclusions and implications of key findings; systematic review registration number. | 1 |
| **Introduction** | | |  |
| Rationale | 3 | Describe the rationale for the review in the context of what is already known. | 2 |
| Objectives | 4 | Provide an explicit statement of questions being addressed with reference to participants, interventions, comparisons, outcomes, and study design (PICOS). | 2 |
| **Methods** | | |  |
| Protocol and registration | 5 | Indicate if a review protocol exists, if and where it can be accessed (e.g., Web address), and, if available, provide registration information including registration number. | Not applicable |
| Eligibility criteria | 6 | Specify study characteristics (e.g., PICOS, length of follow-up) and report characteristics (e.g., years considered, language, publication status) used as criteria for eligibility, giving rationale. | 4 |
| Information sources | 7 | Describe all information sources (e.g., databases with dates of coverage, contact with study authors to identify additional studies) in the search and date last searched. | 4 |
| Search | 8 | Present full electronic search strategy for at least one database, including any limits used, such that it could be repeated. | 4, supplementary data 2 |
| Study selection | 9 | State the process for selecting studies (i.e., screening, eligibility, included in systematic review, and, if applicable, included in the meta-analysis). | 4 |
| Data collection process | 10 | Describe method of data extraction from reports (e.g., piloted forms, independently, in duplicate) and any processes for obtaining and confirming data from investigators. | 5 |
| Data items | 11 | List and define all variables for which data were sought (e.g., PICOS, funding sources) and any assumptions and simplifications made. | 4 |
| Risk of bias in individual studies | 12 | Describe methods used for assessing risk of bias of individual studies (including specification of whether this was done at the study or outcome level), and how this information is to be used in any data synthesis. | 5 |
| Summary measures | 13 | State the principal summary measures (e.g., risk ratio, difference in means). | Not applicable |
| Synthesis of results | 14 | Describe the methods of handling data and combining results of studies, if done, including measures of consistency (e.g., I^2^) for each meta-analysis. | Not applicable |
| Risk of bias across studies | 15 | Specify any assessment of risk of bias that may affect the cumulative evidence (e.g., publication bias, selective reporting within studies). | 5 |
| Additional analyses | 16 | Describe methods of additional analyses (e.g., sensitivity or subgroup analyses, meta-regression), if done, indicating which were pre-specified. | Not applicable |
| **Results** | | | |
| Study selection | 17 | Give numbers of studies screened, assessed for eligibility, and included in the review, with reasons for exclusions at each stage, ideally with a flow diagram. | 5 |
| Study characteristics | 18 | For each study, present characteristics for which data were extracted (e.g., study size, PICOS, follow-up period) and provide the citations. | 5, 6, Table 1 and 2 |
| Risk of bias within studies | 19 | Present data on risk of bias of each study and, if available, any outcome level assessment (see item 12). | 7 |
| Results of individual studies | 20 | For all outcomes considered (benefits or harms), present, for each study: (a) simple summary data for each intervention group (b) effect estimates and confidence intervals, ideally with a forest plot. | 7 |
| Synthesis of results | 21 | Present results of each meta-analysis done, including confidence intervals and measures of consistency. | 7 |
| Risk of bias across studies | 22 | Present results of any assessment of risk of bias across studies (see Item 15). | 7 |
| Additional analysis | 23 | Give results of additional analyses, if done (e.g., sensitivity or subgroup analyses, meta-regression [see Item 16]). | Not applicable |
| **Discussion** | | | |
| Summary of evidence | 24 | Summarize the main findings including the strength of evidence for each main outcome; consider their relevance to key groups (e.g., healthcare providers, users, and policy makers). | 7 -12 |
| Limitations | 25 | Discuss limitations at study and outcome level (e.g., risk of bias), and at review-level (e.g., incomplete retrieval of identified research, reporting bias). | 12 |
| Conclusions | 26 | Provide a general interpretation of the results in the context of other evidence, and implications for future research. | 13 |
| **Funding** | | | |
| Funding | 27 | Describe sources of funding for the systematic review and other support (e.g., supply of data); role of funders for the systematic review. | Not applicable |

**Supplementary table 2. Search strategies and hits based on searches last conducted on May 6, 2020.**

| **Database** | **Search strategy** | **Number hits** |
| --- | --- | --- |
| Pubmed | ((((((((Vaginal microbiota[MeSH Terms]) OR vaginal microbiome[MeSH Terms]) OR vaginal microbiota) OR vaginal microbiome) OR vaginal dysbiosis') OR bacterial vaginosis OR aerobic vaginitis)) AND ((africa[MeSH Terms]) OR sub-saharan africa[MeSH Terms])) AND ((((((pregnant women[MeSH Terms]) OR pregnancy[MeSH Terms]) OR pregnant) OR pregnancy) OR pregnancy outcome[MeSH Terms]) OR pregnancy outcome) | 95 |
| Pubmed | (((((((Vaginal microbiota[MeSH Terms]) OR vaginal microbiome[MeSH Terms]) OR vaginal microbiota) OR vaginal microbiome) OR vaginal dysbiosis') OR bacterial vaginosis)) AND ((((("xxx"[Mesh]) OR (“xxx”) OR (“xxx” women)) AND (((((pregnant women[MeSH Terms]) OR pregnancy[MeSH Terms]) OR pregnant) OR pregnancy) OR (((pregnancy outcome[MeSH Terms]) OR pregnancy outcome)))))) | 207 |
| Embase (Ovid) | (exp Vaginal microbiota/ or exp vaginal microbiome/ or exp vaginal flora/ or (vaginal microbiota or vaginal microbiome or vaginal dysbiosis or bacterial vaginosis or aerobic vaginitis).ti,ab,kw.) AND (exp africa/ or exp Africa south of Sahara/ or sub-Saharan Africa.ti,ab,kw.) AND (exp pregnant women/ or exp pregnancy/ or exp pregnancy outcome/ or (pregnant or pregnancy or pregnancy outcome).ti,ab,kw.) | 148 |
| Cochrane | vaginal microbio* OR genital microbio* in All Text OR "bacterial vaginosis" in All Text OR "aerobic vaginitis" in All Text AND Africa in All Text AND pregnan* in All Text - (Word variations have been searched) | 108 |

At the “xxx” each Sub-Saharan African country was filled in per search, countries selected based on the world bank organization database: Angola, Benin, Botswana, Burkina Faso, Burundi, Cabo Verde, Cameroon, Central African Republic, Chad, Comoros, Congo, Cote D’Ivoire, Equatorial guinea, Eritrea, Eswatini, Ethiopia, Gabon, Gambia, Ghana, Guinea, Guinea-Bissau, Kenya, Lesotho, Liberia, Madagascar, Malawi, Mali, Mauritania, Mauritius, Mozambique, Namibia, Niger, Nigeria, Rwanda, Sao Tome and Principe, Senegal, Seychelles, Sierra Leone, Somalia, South Africa, South sudan, Sudan, Tanzania, Togo, Uganda, Zambia, Zimbabwe (32).

Supplementary Table 3. Questions for the critical appraisal checklist for cohort studies, cross sectional studies, case-control studies, and randomized controlled trial as provided by Joanna Briggs Institute Reviewer’s Manual (64).

| **Question numbers** | **Cohort studies** | **Cross-sectional studies** | **Case-control studies** | **Randomized control trial** |
| --- | --- | --- | --- | --- |
| 1 | Were the two groups similar and recruited from the same population? | Were the criteria for inclusion in the sample clearly defined? | Were the groups comparable other than the presence of disease in cases or the absence of disease in controls? | Was true randomization used for assignment of participants to treatment groups? |
| 2 | 1. Were the exposures measured similarly to assign people to both exposed and unexposed groups? | Were the study subjects and the setting described in detail? | Were cases and controls matched appropriately? | Was allocation to treatment groups concealed? |
| 3 | Was the exposure measured in a valid and reliable way? | Was the exposure measured in a valid and reliable way? | Were the same criteria used for identification of cases and controls? | Were treatment groups similar at the baseline? |
| 4 | Were confounding factors identified? | Were objective, standard criteria used for measurement of the condition? | Was exposure measured in a standard, valid and reliable way? | Were participants blind to treatment assignment? |
| 5 | Were strategies to deal with confounding factors stated? | Were confounding factors identified? | Was exposure measured in the same way for cases and controls? | Were those delivering treatment blind to treatment assignment? |
| 6 | Were the groups/participants free of the outcome at the start of the study (or at the moment of exposure)? | Were strategies to deal with confounding factors stated? | Were confounding factors identified? | Were outcomes assessors blind to treatment assignment? |
| 7 | Were the outcomes measured in a valid and reliable way? | Were the outcomes measured in a valid and reliable way? | Were strategies to deal with confounding factors stated? | Were treatment groups treated identically other than the intervention of interest? |
| 8 | Was the follow up time reported and sufficient to be long enough for outcomes to occur? | Was appropriate statistical analysis used? | Were outcomes assessed in a standard, valid and reliable way for cases and controls? | Was follow up complete and if not, were differences between groups in terms of their follow up adequately described and analyzed?  Were participants analyzed in the groups to which they were randomized? |
| 9 | Was follow up complete, and if not, were the reasons to loss to follow up described and explored? | Not applicable | Was the exposure period of interest long enough to be meaningful? | Were outcomes measured in the same way for treatment groups? |
| 10 | Were strategies to address incomplete follow up utilized? | Not applicable | Was appropriate statistical analysis used? | Were outcomes measured in a reliable way? |
| 11 | Was appropriate statistical analysis used? | Not applicable | Not applicable | Was appropriate statistical analysis used? |
| 12 | Not applicable | Not applicable | Not applicable | Was the trial design appropriate, and any deviations from the standard RCT design (individual randomization, parallel groups) accounted for in the conduct and analysis of the trial? |
| 13 | Not applicable | Not applicable | Not applicable | Not applicable |
